# Effect of Annealing Treatment on Mechanical Properties of Nanocrystalline α-iron: an Atomistic Study

**DOI:** 10.1038/srep08459

**Published:** 2015-02-13

**Authors:** Xuhang Tong, Hao Zhang, D. Y. Li

**Affiliations:** 1 Department of Chemical and Materials Engineering, University of Alberta, Edmonton, AB T6G 2V4, Canada

## Abstract

Claims are often found in the literature that metallic materials can be nanocrystallized by severe plastic deformation (SPD). However, SPD does not generate a well-defined nanocrystalline (NC) material, which can be achieved by subsequent annealing/recovery treatment. In this study, molecular dynamics (MD) simulation is employed to study the effect of annealing on structure and mechanical properties of cyclic deformed NC α-iron, which simulates SPD-processed α-iron. It is demonstrated that grain boundaries in the deformed NC α-iron evolve to a more equilibrium state during annealing, eliminating or minimizing the residual stress. The annealing treatment increases the system's strength by reducing dislocation emission sources, and improves material ductility through strengthening grain boundaries' resistance to intergranular cracks. The results indicate that the annealing treatment is an essential process for obtaining a well-defined NC structure with superior mechanical properties.

Nanocrystalline(NC)^1^ metallic materials have been widely studied due to their superior mechanical, optical, and electrical properties. Severe plastic deformation (SPD)^2^ techniques, e.g., equal channel angular processing (ECAP)^3^, accumulative roll bonding (ARB)^4^, high pressure torsion (HPT)^5^ and ball milling^6^, are applied to reduce the grain size to nano-scale. In the structure generated by SPD, nanoscale grain boundaries (GBs), which play very important roles in determining mechanical properties of NC materials, are mostly in non-equilibrium states^2^ or more rigorously they are diffuse boundaries between dislocation cells. Other defects (e.g., point defects, twinning, stacking fault and porosity) are also commonly observed in SPD structures. These non-equilibrium GBs and defects insides grains may significantly influence the mechanical behavior of nanostructures generated by SPD.

To alter the strength and plasticity of NC metallic materials after SPD, subsequent annealing/recovery treatment is applied^4^,^7^,^8^,^9^,^10^. Moderate annealing without noticeable grain growth, i.e., low temperature and short annealing time, was reported to increase the strength of SPD NC materials^4^,^9^,^10^. This phenomenon, termed as “annealing hardening”^4^, is opposite to the situations of traditional coarse-grained metals, where annealing generally lowers the strength by reducing dislocation density. Several possible mechanisms are proposed to explain this phenomenon. During annealing, the dislocation density decreases, resulting from annihilation of dislocations with opposite Burger's vectors^11^ and dislocation absorption at grain boundaries. On the nanoscale, a system with dislocations starvation may have higher strength^12^. Besides, it has been shown that after annealing, GBs reach more equilibrium state and become sharper^13^. GBs in more equilibrium states increase the barrier to dislocation emission^4^,^14^ and GB sliding^9^, corresponding to a higher overall yield strength.

Studies on the effects of annealing on NC metals' ductility reported in literature are however not always consistent. The ductility of NC Ti (with grain size about 120 nm) processed by high pressure torsion was reported to be improved by 30% after annealed at 300°C for 10 min^9^. A similar trend was observed in electrodeposited NC Ni (with grain size about 30 nm) after annealed at 100°C^10^. However, it was also reported that annealing at 150°C markedly lowered the ductility of NC Al (with grain size about 200 nm) produced by accumulative roll bonding^4^, which is explained that annealing reduces the dislocation density and fewer dislocations are available to conduct plastic deformation. In the above-mentioned studies, though the materials and annealing parameters are different, the main purpose of performed moderate annealing is the same, i.e., to re-arrange defects and GBs towards more equilibrium states without grain growth. The inconsistency could be caused by some un-controllable factors, e.g., impurity segregation^10^,^15^ and possible precipitation which may occur during annealing. In Ref. 10, the sulfur concentration at grain boundaries in NC Ni could reach up to 6.3 wt. % after annealing, which made the NC Ni exhibit more brittle characteristics. In previous computational studies^7^,^14^, artificially induced non-equilibrium GBs and relatively small grain size (12 nm, which is close to the region with inverse Hall-Petch relationship^16^) may complicate the situation with extra factors, such as grain boundary sliding and grain rotation. These discrepancies are also an indication that there lacks a thorough understanding about the intrinsic effects of annealing on mechanical properties of NC metals, especially for the less studied BCC metals. Furthermore, the ductility of NC metals, which is represented by the maximum plastic strain at failure during tensile test, can be largely affected by nano cracks/voids nucleation, growth and coalescence^17^,^18^. The role of fracture was not taken into account in previous studies regarding the effect of annealing on NC metals. To clarify mechanisms responsible for the effects of annealing on NC metals, we conducted a molecular dynamics (MD) simulation study. The main objectives of this study are to investigate effects of annealing on strength and ductility of deformed NC materials and demonstrate that annealing/recovery treatment is an essential step to turn a SPD microstructure into a well-defined nanocrystalline one with superior mechanical properties.

A polycrystalline α-iron with grain size ranging from 18 nm to 23 nm was generated from Voronoi construction^19^, shown in Fig. 1a. The choice of α-iron is attributed to its industrial importance and well-established database. In order to simulate non-equilibrium nanoscale GBs and defects caused by SPD, cyclic plastic deformation in y-direction was applied to the NC α-iron (stress-strain curves shown in supplementary Fig. S1). Thereafter, two sample systems were created, one was in as-processed state and the other was annealed. For the annealed system, it was relaxed at 750 K for 1.6 ns followed by cooling to 300 K. Due to the limited annealing time in MD simulation, annealing temperature was set to be higher than that of moderate annealing in experimental study. In the annealed system, no grain growth was observed. Simulation details are described in **Methods** section.

## Results

### Voronoi volume and residual stress

We calculated the Voronoi volumes, hydrostatic pressures and stress tensors in y- direction of each atom in as-processed and annealed systems. Before determining the Voronoi volume of each atom, energy minimization was carried out using a conjugate gradient algorithm until the force on every atom was smaller than 10^−8^ eV/Å. After the energy minimization, the Voronoi volume of each atom was calculated using Voro++^20^. As atoms' fluctuations around the lattice sites may result in very high local stress, a time-average procedure was performed while calculating local stress. The stresses of each atom were recorded every 4 femtoseconds within 100 femtoseconds, and then averaged to eliminate the influence of atom fluctuation. The stress tensor (σ_yy_) was calculated in *virial* form. The hydrostatic pressure was represented by −(σ_xx_ + σ_yy_ + σ_zz_)/3.

**Figure 1:**
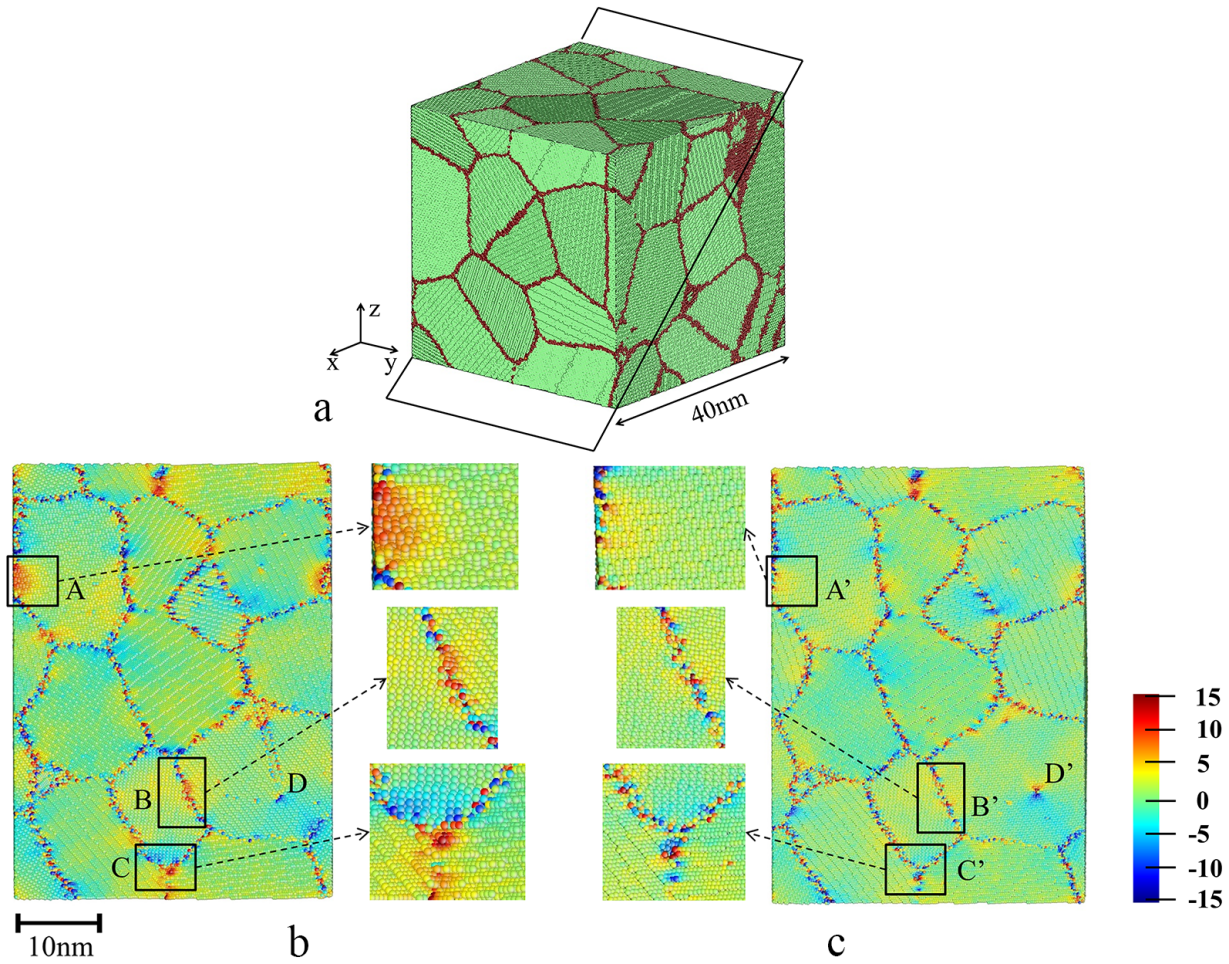
System configuration (a) and cross-section plots of residual stress (σ_yy_) in (b) as-processed and (c) annealed systems. The polycrystalline system is built with 3D periodic boundary condition to simulate bulk NC α-iron. The plane in (a) sketched by black lines is the plane of cross-section views displayed in (b) and (c). The pressures in x-, y- and z- directions are 0 bar and the temperature is 300 K. Atoms are colored by σ_yy_; color map is in unit of GPa. Comparisons between (b) and (c) indicate that annealing treatment has reduced the internal stress, driven grain boundaries and triple junctions to more equilibrium states and converted a deformation twinning(region D in (b)) to a <111>{112} edge dislocation(region D′ in (c)).

Figure 1 shows cross-sectional views of as-processed system (b) and annealed one (c), in which atoms are colored by their normal stresses in y- direction (σ_yy_). Comparing Fig. 1b with Fig. 1c, the stress distribution in the annealed sample is more homogenous than that in the as-processed sample. For instance, the high local stress in region A of the as-processed sample was markedly reduced by annealing, as shown in region A′. Clearly, the annealing drove the system closer to a more equilibrium state with reduced local stress and ordered atomic structures [also see those shown in regions B and B′ (GB), C and C′ (triple junction) in Fig. 1b and c, respectively]. Annealing also turned a <111>{112} twinning in as-processed sample (region D) into a stable <111>{112} edge dislocation (region D′). This conversion of the planar defect to a linear defect may also decrease the system's energy^21^.

**Figure 2:**
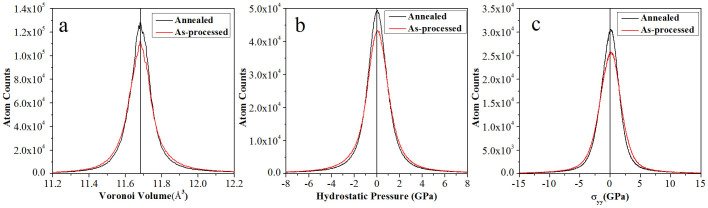
Statistical distributions of (a) Voronoi volume, (b) hydrostatic pressure and (c)stress tensor in y-direction of each atom in both annealed and as-processed systems. These histograms quantitatively demonstrate more ordered atomic structures (represented by Voronoi volume) as well as reduced residual stress in the annealed system.

Figure 2 illustrates statistical distributions of calculated Voronoi volume, hydrostatic pressure and stress tensor in y-direction (σ_yy_) of each atom in both systems. At 300 K, the equilibrium Voronoi volume of a α-iron atom in perfect BCC structure is 11.68 Å^3^. Figure 2a shows that Voronoi volumes of more atoms are close to 11.68 Å^3^ in the annealed system than in the as-processed system. In Fig. 2b, the distribution of hydrostatic pressure of each atom shows the same trend, i.e., more atoms in annealed sample are in states closer to the equilibrium one (0 GPa). The distribution of stress tensor in y- direction (σ_yy_) is consistent with hydrostatic pressure distribution, as shown in Fig. 2c. Note that the overall system stress is 0 GPa in all three directions; ideally most atoms should also have local stress at the level of 0 GPa. However, as illustrated in Fig. 2b and c, residual stresses still exist, caused by the experienced cyclic loading. The local residual stress can reach up to 20 GPa. In NC metallic materials with abundant GBs, local atomic structures and local stress fluctuations at GB areas play crucial roles in determining materials' overall strengths^21^,^22^,^23^. The different atomic configurations and residual stress distributions of annealed and as-processed system should influence their overall mechanical properties.

### Tensile deformation test

In order to evaluate mechanical properties of as-processed and annealed samples, uniaxial tensile deformation with a strain rate of 10^−1^ ns^−1^ at 300 K was applied to both the systems in y-direction until failure (the time interval is 2 femtoseconds). During deformation, the pressure in x- and z- directions was kept at 0 bar by NPT ensemble. The ductility of each system was measured, which was the plastic strain at failure.

**Figure 3:**
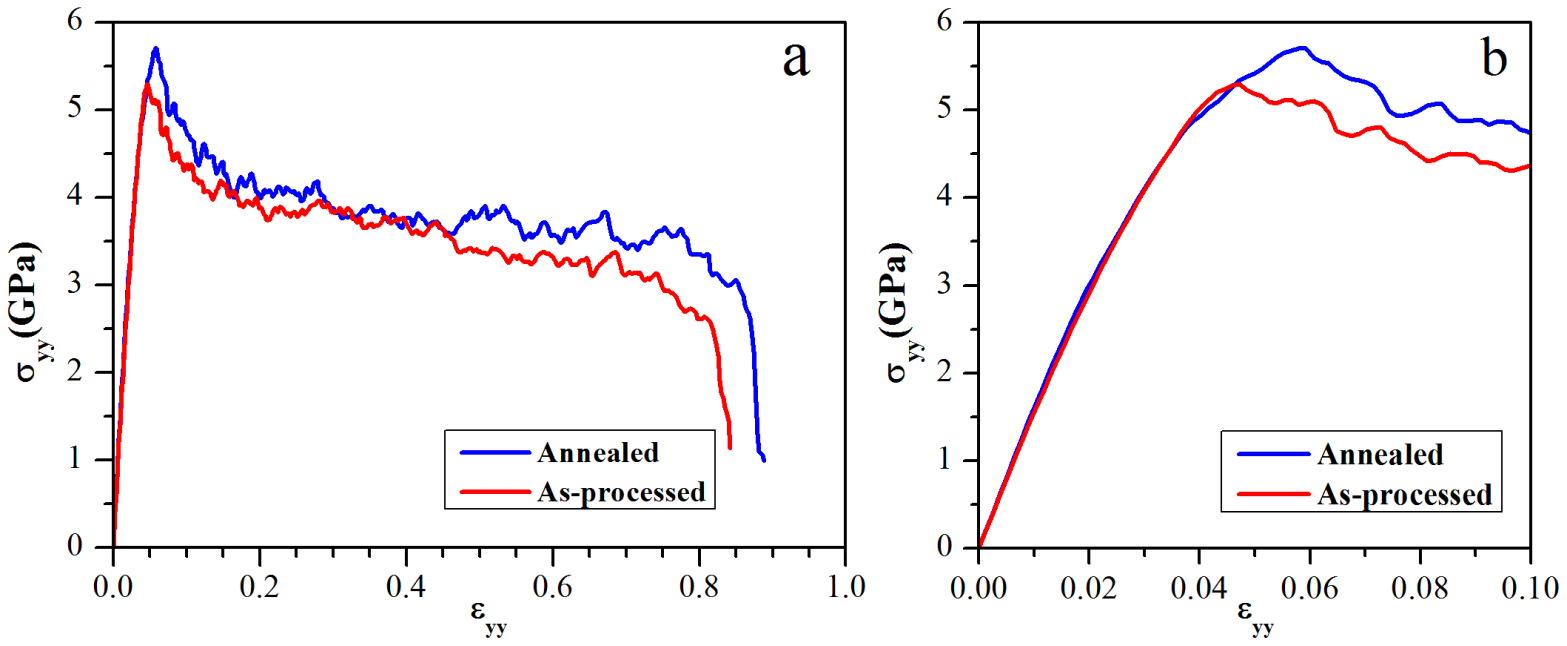
Stress-strain curves of (a) tensile deformation applied to as-processed sample and annealed sample from no strain to failure, and (b) enlarged stress-strain curves of an early deformation stage up to 10% strain. Compared with as-processed system, the annealed sample demonstrates better strength as well as better ductility.

Stress-strain curves of uniaxial tensile deformation in y- direction are illustrated in Fig. 3a and b. As shown, the annealing treatment increased the maximum stress and flow stress of NC α-iron, which is consistent with experimentally observed “anneal hardening”. Besides, the ductility of NC α-iron was also improved by annealing, i.e., the failure strain is increased from 80% to 86%. Corresponding values of the mechanical properties are presented in Table 1. In experimental studies, the nominal overall fracture strains of NC metals are less than the values obtained from the simulation. This could be attributed to the fact that experimentally the fracture zone often experiences very large true strain. For instance, grains in shear bands in NC Fe are deformed to true strain of 2–3 when the overall strain is 14%^24^.

### Dislocation emission during yielding

The overall deformation behavior (mainly the plastic deformation) is directly related to dislocation activities. When the grain size is below 100 nm but above 10 nm (without the inverse H-P relation), dislocation emission from grain boundaries largely contributes to plastic deformation, strongly affecting the yield strength and maximum strength of a system. Dislocation emission is a highly localized behavior, and the internal stress concentrators at grain boundaries in bulk NC metallic materials could act as dislocation emission source^23^.

**Figure 4:**
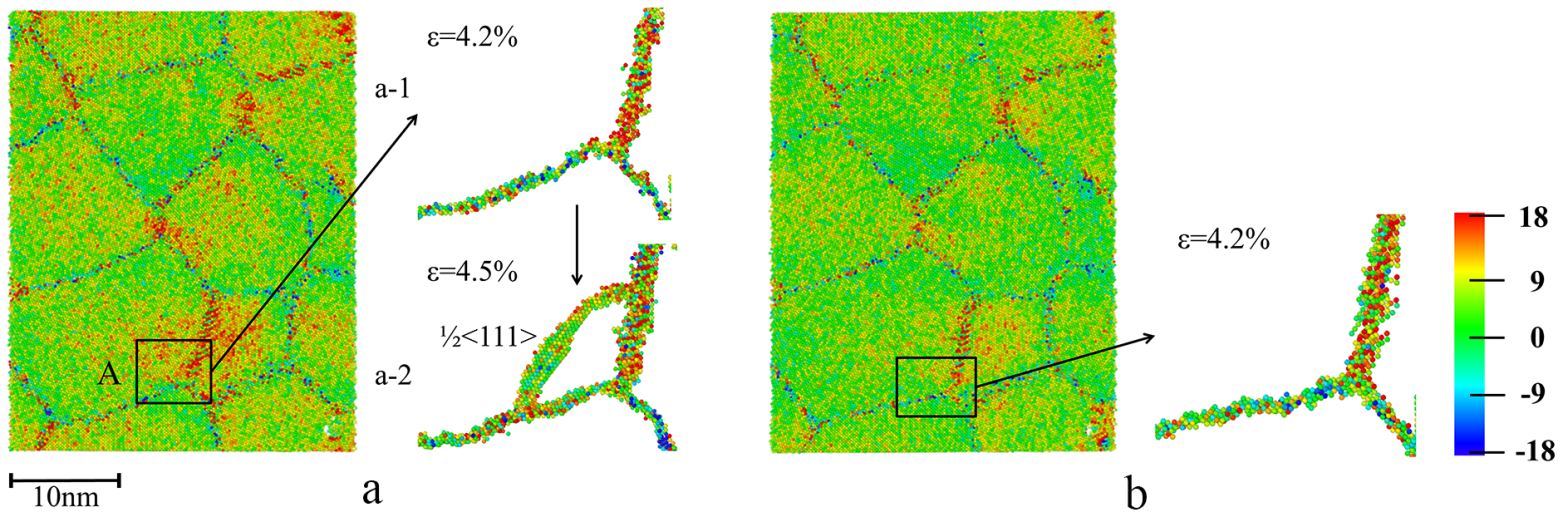
The cross-section views of (a) as-processed sample and (b) annealed sample at 4.2% strain. Atoms are colored by stress tensor in y- direction (σ_yy,_). Color map is in the unit of GPa. The high local stress of region A in as-processed sample (a) at the strain of 4.2% (a-1) results in emission of a ½<111> dislocation at the strain of 4.5% (a-2). At the strain of 4.2%, local stress in the same region in annealed sample (b) is more homogeneous, and no dislocation is emitted from this region when deformed further.

In this study, the maximum strength of the annealed system is 8% higher than that of as-processed one, as shown in Fig. 3b and Table 1. The as-processed sample reaches its maximum strength of 5.30 GPa at 4.6% strain. The local stress distribution of the as-processed sample before reaching the maximum strength was analyzed. Figure 4a shows a cross-sectional view of stress (σ_yy_) distribution in the as-processed sample. Figure 4a-1 is an enlarged view of a triple junction region (marked by “A”), where high local stress is present at 4.2% strain. When the system was further deformed from the strain of 4.2% to 4.5%, a ½<111> dislocation emitted from the triple junction, as shown in Fig. 4a-2. Figure 4b shows the cross-sectional view of stress distribution in the same location in the annealed sample when it was deformed to a strain of 4.2%. As shown, the local stress is less inhomogeneous, and no dislocation was emitted when the strain was increased to 4.5%. The local stress analysis verifies that annealing treatment reduces the amount of active sources for dislocation emission, thus strengthening the material. Larger stress is required in order to cause yielding behaviors, i.e., dislocation emission, in the annealed system.

As annealing treatment strengthens NC α-iron by reducing the amount of dislocation emission sources, it is reasonable to expect that plastic deformation can weaken NC α-iron as a result of introducing various defects and driving GBs away from the equilibrium state, both of which facilitate dislocation emission. This may also be seen from the strength of the initially constructed defects-free system (σ_max_ = 5.54 GPa, “1^st^ tension” curve in supplementary Fig.S1), which is higher than that of the cyclically deformed one (σ_max_ = 5.30 GPa, see “as-processed” curve in Fig. 3b).

Although GBs in annealed sample are less easy to emit dislocations, as the system is further deformed with an increase of the applied stress, other dislocation sources can be activated. Dislocation emission determines the system's maximum strength and also influences further plastic deformations and consequently the system's ductility.

### Ductility

**Figure 5:**
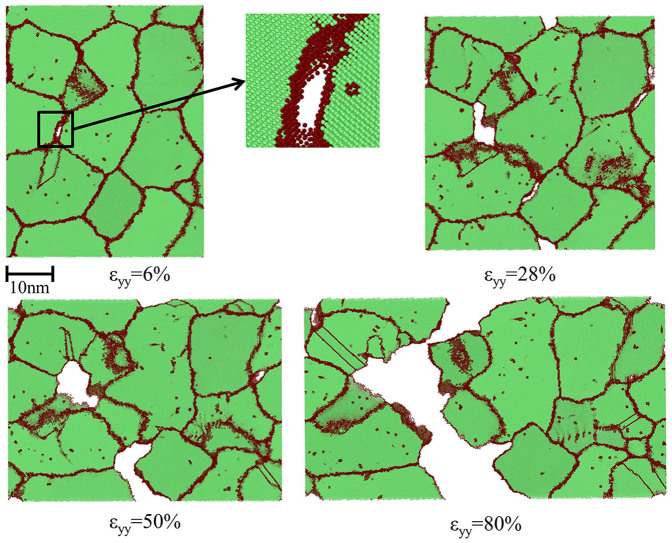
Cross-section views of atomic configurations of the as-processed system during tensile deformation. Atoms are colored by CNA values, while red spheres represent GB and dislocation atoms, green spheres are BCC atoms. As the strain increases, the deformation process includes voids/cracks nucleation, growth and coalescence and eventual failure at 80% strain. The annealed system has the same failure mode of intergranular cracking with the as-processed system.

The ductility is the plastic strain at fracture, which is directly related to the fracture behavior. For BCC^25^ and FCC^18^,^26^ materials, simulation studies show that fracture of NC materials is mostly in the intergranular failure mode. In BCC structures without truly close-packed planes, nucleation and growth of intergranular cracks/voids are alternative ways to relieve strain energy during tensile deformation, especially at large strains. The cross-sectional views in Fig. 5 show the process of crack nucleation, growth, coalescence and system failure in as-processed sample during tensile deformation. The annealed system has the same failure mode of intergranular cracking with the as-processed system.

To quantitatively analyze the fracture process, we propose a simple method to calculate changes in the volume fraction of cracks (i.e., the ratio of the crack volume to the total volume of the system) during deformation. As 3D periodic boundary condition was applied to the system, identified atoms with large Voronoi volumes can be considered to be associated with nano cracks/voids inside the system. At 300 K and 0 bar, the Voronoi volume of a perfect BCC atom is 11.68 Å^3^. If one atom is associated with a nano void or crack, the Voronoi volume of that atom will be significantly larger than that of perfect BCC atom or atoms belonging to dislocations and GBs. In a perfect single crystal, if we delete several atom layers to generate a crack, of which the energetic state is close to that of free surface, then the Voronoi volume of crack atoms can be applied to identify voids and cracks in polycrystalline materials. For the Mendelev potential applied in this study, the Voronoi volume of {100} crack surface atom is 28.8 Å^3^, while 23.3 Å^3^ for {110} crack surface atoms and 33.3 Å^3^ for {111} crack surface atoms. Here we choose the average value (28.5 Å^3^) as a critical value to determine whether an atom is at crack surface. Any atom with Voronoi volume higher than critical value is identified as a void/crack atom and volumes of such atoms are summed up as the total crack volume inside the system.

**Figure 6:**
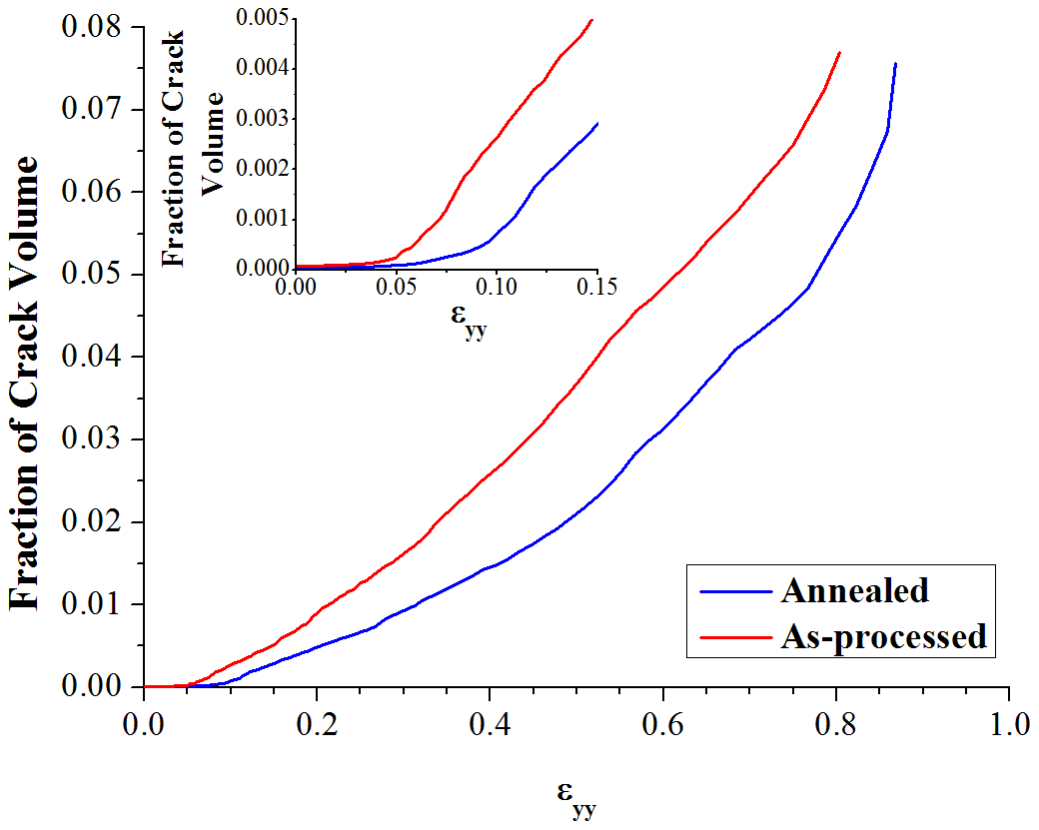
Evolution of crack fraction (crack volume/system volume) with tensile strain for annealed and as-processed samples. The inset plot shows details in the early deformation stage with the strain ranged from 0 to 15%. The annealed sample shows a larger resistance to intergranular cracking than as-processed sample.

Calculated volume fractions of cracks as a function of strain for both annealed and as-processed samples are shown in Fig. 6. The inset plot in Fig. 6 shows changes in the crack fraction in the early deformation stage before 15% strain. Compared with that in the annealed sample, crack/void nucleation occurred in the as-processed sample at smaller applied strains. The volume fraction of cracks/voids in the as-processed sample is always larger than that of the annealed sample at the same strain level. As the GBs in annealed sample have been equilibrated by annealing, they show higher resistance to intergranular cracking. The difference in crack fraction between the annealed and as-processed samples shown in Fig. 6 well explains why the annealed sample's ductility is 7.5% higher than that of the as-processed sample.

## Discussion

In this study, annealing treatment is demonstrated to alter the structure of SPD-processed NC α-iron, i.e., reduce defect density and equilibrate GBs' energetic states without recrystallization and grain growth. In experimental studies, such atomistic structural evolution can be achieved by carefully tuning the annealing temperature, duration and other relevant parameters. For instance, defect minimization without grain growth in NC metallic materials during annealing treatment have been observed in NC aluminum^4^, titanium^9^, nickel^10^, copper-nickel alloys^13^ and nanocrystallized stainless steel^27^. Typical annealing temperatures employed in these studies are around 0.2 ~ 0.3 *T*_m_ (melting temperature) to avoid grain growth. In NC pure α-iron, grain size was stabilized around 18 nm after annealed at 0.26 *T_m_*^28^ and no grain growth was observed at the room temperature due to lack of sufficient thermal energy. The defect ordering or minimization and GBs equilibration benefit NC materials. The present computational study has clearly demonstrated that the low-temperature annealing or recovery treatment improves the mechanical properties of SPD-processed α-iron.

Deformation of NC metallic materials involves various possible mechanisms, e.g., grain rotation^29^, GB sliding^30^, GB diffusion^31^, dislocation emission^32^,^33^, twinning^21^,^34^, cavitation^18^,^26^ at GBs, etc. Previous studies regarding the effects of annealing treatment are mainly focused on dislocation emissions from grain boundaries^4^,^7^,^14^. However, competition and synergy between different deformation mechanisms are important for understanding NC materials' mechanical properties^18^. There could be a single dominating mechanism or a mixture of multiple mechanisms, determined by lattice structure, atomic bond strength, grain size^32^, strain level^18^, strain rate^35^, deformation temperatures^36^, etc. In the present α-iron polycrystalline system with grain size about 20 nm, we observed a strong dependence of deformation mechanisms on the strain level. In the early deformation stage, the yield strength and maximum strength of the system are mainly determined by dislocation emission^32^ from grain boundaries. Due to the relatively low energy barrier of <111>{112} twinning compared with <111>{112} dislocation^37^, deformation twinning is also observed. Given the fact that the internal stress concentrations at grain boundaries could facilitate yielding process and determine the overall strength^23^, the beneficial effect of grain boundary equilibrations on NC metals' strength can be expected. We have explicitly demonstrated that the annealed system is strengthened by the reduced amount of dislocation/twinning emission sources. After a certain amount of plastic strain (6% ~ 8% engineering strain) is introduced, intergranular voids/cracks start to nucleate at grain boundaries. In BCC metals without truly close-packed planes, formation of intergranular voids/cracks provides an alternative path for relaxing the strained system. Intergranular voids/cracks grow and coalesce when the system is further deformed. In this case, the deformation involves a combination of dislocation/twinning emission from GBs and intergranular voids/cracks nucleation and growth. As the strain continuously increases, intergranular cracking becomes predominant before the NC α-iron's eventually fails. The method proposed for the study quantitatively characterizes intergranular cracking process and demonstrates that annealed NC α-iron has increased resistance to intergranular cracking. As for other factors which influence NC α-iron's mechanical properties, e.g., strain localization^24^, strain rate and deformation temperature, they would be included in our follow-up studies.

In conclusion, we investigated the effects of annealing on the mechanical properties of NC α-iron and relevant mechanisms. The moderate annealing or recovery treatment diminishes defects, eliminates or minimizes the residual stress and drives GBs towards the equilibrium state. As a result, the annealing treatment not only increases the strength of the NC α-iron by reducing dislocation emission sources but also improves its ductility through strengthening the GBs' resistance to intergranular cracking. The improvement in ductility of the NC material by annealing demonstrated in this computational study is an indication that those experimental observations of annealing-induced loss of ductility in NC materials, reported in the literature, should not be an intrinsic phenomenon and could be attributed to other possible factors, such as impurity segregation or strain localization at grain boundaries. Besides, this study also indicates that annealing treatment is an essential step for obtaining a well-defined NC structure with superior mechanical properties.

## Methods

Voronoi construction^19^ was applied to build the polycrystalline system. In a cubic box having a size of 40 × 40 × 40 nm^3^ and 3D periodic boundary conditions, 27 “seeds” were randomly distributed. Grains with random misorientations were generated from the “seeds” and filled the box with about 5.5 million atoms. The grains had their sizes in the range from 18 to 23 nm. For convenient dislocation analysis, the misorientations between adjacent grains were controlled to be larger than 15° in order to avoid low-angle grain boundaries (LAGBs). EAM potential was used to represent the interatomic force of α-iron, developed by Mendelev *et al.*^38^ The generalized stacking fault energy, interstitial and vacancy formation energy, thermal expansion and dislocation properties predicted by this potential are in good agreement with DFT data^39^, which makes it effective in simulating tensile deformation of polycrystalline α-iron^37^,^40^. All MD simulations were carried out with LAMMPS^41^.

MD simulations were conducted using isothermal-isobaric (NPT) ensemble via Nose-Hoover thermostat^42^,^43^. The initially constructed system was relaxed at 700 K for 500 picoseconds (the time interval is 1 femtosecond) to reach more stable GB structures. The model system was then cooled to 300 K at a cooling rate of 2 K/picoseconds; during the cooling progress, pressures in x-, y- and z- directions were controlled at 0 bar. The relaxed system had stable grain boundary structures and was free with defects inside each grain. In order to mimic a SPD progress, cyclic loading in y-direction was applied to the polycrystalline system. Tensile and compress deformations were introduced during cyclic loading at a strain rates of 10^−1^ ns^−1^. The system was first pulled to reach a strain of 8% and then compressed to a strain of −8%, followed by a final tensile process up to 6% strain to avoid porosity inside the system. Corresponding stress-strain curves are shown in supplementary Fig. S1. Compared to the other artificially generated GBs^7^,^14^, the atomic structure in present simulated NC system experienced plastic deformation could be closer to the configuration of NC α-iron generated by SPD.

After the above-mentioned cyclic loading, sample systems were kept at 300 K and 0 bar for 200 picoseconds. Two sample systems were created, one was in as-processed state and the other was annealed. For the annealed system, it was relaxed at 750 K for 1.6 ns followed by cooling to 300 K. The time interval was 2 femtoseconds. Due to the limitation of simulation time, the annealing temperature in MD simulation was set to be higher than those in experimental studies, so that more atomic ordering could be achieved within the simulation time without grain coarsening. Such computational annealing treatment reflects experimental low-temperature annealing (typically around 0.2 ~ 0.3 T_m_) or recovery treatment^10^,^13^,^27^,^28^. In order to test the mechanical properties, uniaxial tensile deformations with a strain rate of 10^−1^ ns^−1^ at 300 K were applied to both the systems in y-direction until failure. During uniaxial tension, the pressure in x- and z- directions was kept at 0 bar. Systems were visualized using Ovito^44^ and AtomEye^45^. All simulations were carried out in the Bugaboo Dell Xeon clusters provided by WestGrid.

## Author Contributions

H.Z. and D.Y.L. developed the idea and designed the simulation. X.T. performed MD simulations. X.T., H.Z. and D.Y.L. analyzed the data and wrote the manuscript.

## Supplementary Material

Supplementary Information Supplementary Figure

## Figures and Tables

**Table 1:** Mechanical properties of annealed and as-processed systems. σ_max_, the maximum stress during tensile deformation; σ_flow(ε < 0.2)_, the flow stress before 20% strain; σ_flow(ε > 0.2)_, the flow stress from 20% strain to failure; ε_failure_, the plastic strain at failure and K, the toughness

	σ_max_(GPa)	σ_flow(ε < 0.2)_(GPa)	σ_flow(ε > 0.2)_(GPa)	ε_failure_	K(GPa)
Annealed	5.72	4.57	3.70	0.86	3.31
As-processed	5.30	4.31	3.47	0.80	2.89
