# Supplementary material for: Effect of Annealing Treatment on Mechanical Properties of Nanocrystalline α-iron: an Atomistic Study
Source: Sci Rep. 2015 Feb 13;5:8459. doi: 10.1038/srep08459 (PMC4327417; doi:10.1038/srep08459)
Supplement: Supplementary Information — Supplementary Figure [file srep08459-s1.pdf]

Effect of Annealing Treatment on Mechanical Properties of Nanocrystalline α-iron: an Atomistic Study

Xuhang Tong, Hao Zhang, D. Y. Li

Supplementary Information

Supplementary Figure S1

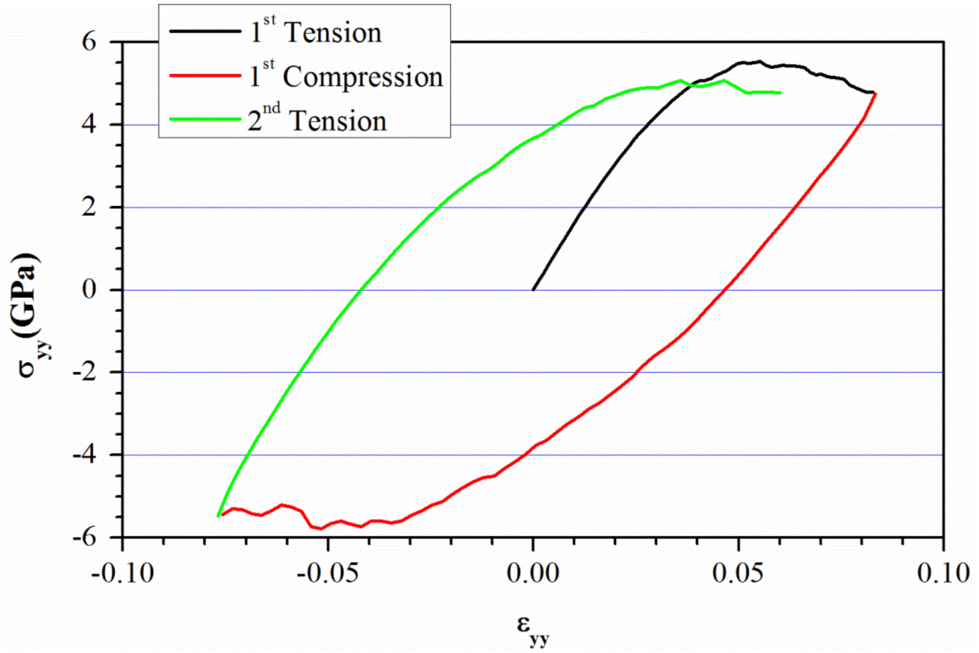

Stress-strain curves of the system subjected to cyclic load in y- direction. The temperature was kept at 300k and the pressures in x- and z- direction were controlled at 0 bar by NPT ensemble. The system was first pulled to reach a strain of 8% and then compressed to a strain of -8%, followed by a final tensile process up to 6% strain to avoid porosity inside the system. The true strain rate of 1st and 2nd tension was 10^{-1} ns^{-1}, and -10^{-1} ns^{-1} for 1st compression.
